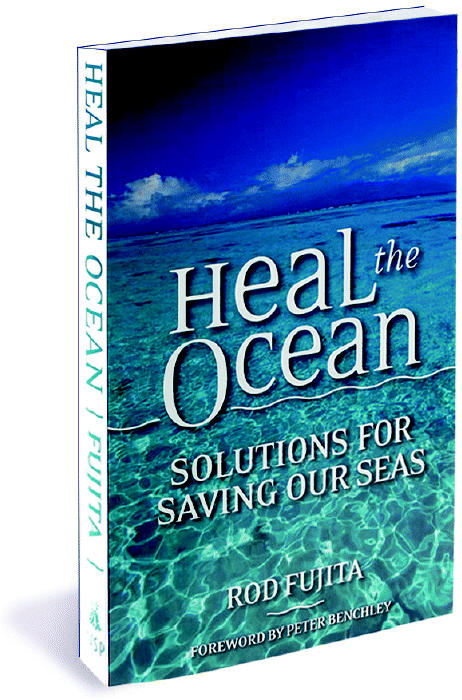# Heal the Ocean: Solutions for Saving Our Seas

**Published:** 2004-11

**Authors:** Paul A. Sandifer

**Affiliations:** Paul Sandifer is a member of the U.S. Commission on Ocean Policy, a former director of the South Carolina Department of Natural Resources, and currently senior scientist for the National Centers for Coastal Ocean Science within NOAA’s National Ocean Service. He is located at the Hollings Marine Laboratory in Charleston, South Carolina.

By Rod Fujita

Gabriola Island, BC, Canada:New Society Publishsers, 2003. 227 pp. ISBN: 0-86571-500-9, $16.95 paper

In only 198 pages of text, Rod Fujita’s *Heal the Ocean: Solutions for Saving Our Seas* covers major threats to the health of the coast, nearshore waters, coral reefs, the continental shelf, and the deep sea. Fujita writes convincingly and from personal experience about the sickening of our coastal waters and the planetary ocean, and he blames such impacts squarely on humans. Part diagnosis, part prescription, part lecture, and part pep talk, *Heal the Ocean* is never boring, although occasionally irritating. However, this is not a balanced view of the issues or a scientific review, but an advocacy piece, and a good one based on Fujita’s extensive experience as a senior scientist at Environmental Defense.

Fujita argues that there are major and growing threats to the health of our coastal and ocean waters and that society must take steps now to stave off potentially catastrophic impacts in the marine environment. Similar findings and concerns have been trumpeted loudly and repeatedly at high levels in the United States recently in the reports and numerous public statements of two independent bodies, the Pew Ocean Commission and the U.S. Commission on Ocean Policy. Never before have there been such broad-based and clear calls for action to address humans’ impacts on marine ecosystems and for the development of a strong ocean stewardship ethic in the American public.

Relying heavily on West Coast examples and issues where Environmental Defense played a lead role, Fujita briefly but thoughtfully describes several major environmental issues, including the “dolphin-safe tuna” and sound-in-the-sea controversies, among others. He also takes a useful look at new threats to deep ocean vent communities and related environments.

A recurring theme is that the most practical way to address many of the ocean’s ills is to create a nationwide network of marine reserves. Fujita discusses the very human processes and divergent opinions involved in developing several protected areas, including the Bonaire Marine Park, the Florida Keys Marine Sanctuary, the Florida Bay Restoration effort, and the Northwest Hawaiian Islands coral reef reserve. Although “marine reserves” or “marine protected areas” may connote different things to different people, Fujita argues persuasively, though not exclusively, for no-take reserves and that a system of carefully chosen and completely protected areas would provide substantial benefits for a broad range of marine species and habitat types. These assertions would have benefited from a more comprehensive review of the scientific literature.

Although many actors appear in Fujita’s drama, his principal antagonists in the battle for the future of the oceans appear to be the organized environmental community—which he sees as the appropriate group to decide what is sustainable and what is not—and fisheries, especially fishery managers. A glaring weakness is that the book provides no perspective from the fishery community, particularly managers, despite the major criticisms Fujita levels at them. In my opinion, the United States has many highly principled, scientifically grounded, and courageous fishery managers who are dedicated to managing sustainable - fisheries and recovery of overexploited populations. Unfortunately, it appears Fujita never met any of them.

Yet the strengths of Fujita’s book lie not in his recitation of an ever-expanding catalog of injuries or the casting of blame, but rather in his message of hope based on some practical suggestions and the optimistic view (which I share) that the American people can become educated to the types and extent of problems and then take necessary actions. Besides marine reserves, he suggests ideas about molding and mobilizing public opinion, managing mining in the deep sea to prevent some environmental impacts, managing fisheries more sustainably, and using renewable resources such as wind, tidal, and thermal energy to - produce electrical power, at least at small scales.

Those wishing to stimulate thinking about the very real problems facing our ocean environments and potential solutions will find Fujita’s - interesting and eminently readable book a very good place to start. But serious students of these topics will need much more information.

## Figures and Tables

**Figure:**